# Design and fabrication of a semi-transparent solar cell considering the effect of the layer thickness of MoO_3_/Ag/MoO_3_ transparent top contact on optical and electrical properties

**DOI:** 10.1038/s41598-021-92539-8

**Published:** 2021-06-22

**Authors:** Çağlar Çetinkaya, Erman Çokduygulular, Barış Kınacı, Feyza Güzelçimen, Yunus Özen, Halil İbrahim Efkere, İdris Candan, Serkan Emik, Süleyman Özçelik

**Affiliations:** 1 Physics Department, Faculty of Science, Istanbul University, 34134 Istanbul, Turkey; 2 Graduate School of Engineering and Science, Istanbul University, 34116 Istanbul, Turkey; 3 Department of Engineering Sciences, Faculty of Engineering, Istanbul University-Cerrahpaşa, 34320 Istanbul, Turkey; 4 Department of Physics, Faculty of Science, Gazi University, 06500 Ankara, Turkey; 5 Photonics Application and Research Center, Gazi University, 06500 Ankara, Turkey; 6 Deparment of Metallurgical and Materials Engineering, Faculty of Technology, Gazi University, 06500 Ankara, Turkey; 7 Department of Physics, Kocaeli University, 41001 İzmit, Kocaeli Turkey; 8 Department of Chemical Engineering, Faculty of Engineering, Istanbul University-Cerrahpaşa, 34320 Istanbul, Turkey; 9 Department of Photonics, Faculty of Applied Sciences, Gazi University, 06500 Ankara, Turkey

**Keywords:** Solar energy and photovoltaic technology, Metals and alloys, Solar cells

## Abstract

We conducted the present study to design and manufacture a semi-transparent organic solar cell (ST-OSC). First, we formed a transparent top contact as MoO_3_/Ag/MoO_3_ in a dielectric/metal/dielectric (DMD) structure. We performed the production of an FTO/ZnO/P3HT:PCBM/MoO_3_/Ag/MoO_3_ ST-OSC by integrating MoO_3_/Ag/MoO_3_ (10/$d_{m}$/$d_{{od}}$ nm) instead of an Ag electrode in an opaque FTO/ZnO/P3HT:PCBM/MoO_3_/Ag (–/40/130/10/100 nm) OSC, after theoretically achieving optimal values of optical and electrical parameters depending on Ag layer thickness. The transparency decreased with the increase of $d_{m}$ values for current DMD. Meanwhile, maximum transmittance and average visible transmittance (AVT) indicated the maximum values of over 92% for $d_{m} ~$ = 4 and 8 nm, respectively. For ST-OSCs, the absorption and reflectance increased in the visible region by a wavelength of longer than 560 nm and in the whole near-infrared region by increasing $d_{m}$ up to 16 nm. Moreover, in the CIE chromaticity diagram, we reported a shift towards the D65 Planckian locus for colour coordinates of current ST-OSCs. Electrical analysis indicated the photogenerated current density and AVT values for $d_{m} = 6$ nm as 63.30 mA/cm^2^ and 38.52%, respectively. Thus, the theoretical and experimental comparison of optical and electrical characteristics confirmed that the manufactured structure is potentially conducive for a high-performance ST-OSC.

## Introduction

Organic solar cells (OSC), where organic materials form the active region, are very useful in terms of low-cost production and fabrication processes, flexibility, lightness and roll-to-roll printability^1–5^. Though in the past, the power conversion efficiency (PCE) values of inorganic semiconductor-based solar cells were much higher than OSCs, today, the PCE of OSCs has raised over 18%^6,7^. In addition, many studies have been conducted to improve PCE, such as the adoption of solvent additives, the use of various active layers, the use of thermal or solvent annealing processes and the use of the triple strategy and reverse structure cells^7–13^.

The optical band gap of organic semiconductor materials can be easily tuned. The thickness of organic active layers in OSC-based photovoltaic technology is about one hundred nanometres for adequate photon harvesting. The ability to form structures, which are thin and have high absorbance, has created the potential to design a semi-transparent organic solar cell (ST-OSC), which has photon harvesting in the near-infrared range and transmittance in the visible light region. When the necessary adjustment and modification is made in the ST-OSC structure, the transparency can be effectively achieved in the visible region (VR) of the solar spectrum as well as it can also absorb light in the NIR region^7^. Thus, the use of ST-OSCs enables the design of state-of-the-art window and curtain applications that can convert sunlight in order to generate electrical power.

For greenhouse applications, because chlorophylls function in a small range of the light spectrum, ST-OSCs can be adjusted to be highly transparent over the spectrum that is required for plant growth^14–16^. Especially in the building industry, building-integrated photovoltaic (BIPV) technology is becoming one of the most effective techniques to power buildings with renewable energy^14,17^. ST-OSCs’ translucence not only provides them to operate as a generator but also reduces electricity consumption by allowing natural light to pass through^17^. The application of ST-OSCs on windows in buildings can enable social living areas with smart and healthy home technologies to receive sunlight.

The top contact material used in inverted OSC is made with thick metal electrodes, such as Ag, Au, and Al, which are highly reflective in VR^18^. Regardless of the optical transmittance and reflection properties of the polymer structure forming the active region, the transmittance characteristic of the OSC is determined by the top contact. Hence, the structure becomes completely opaque. One of the most vital steps of ST-OSC design is to make a top contact with high conductivity and optical transmittance, especially in the VR. In the meanwhile, double-sided transparent solar cells enable light response in optoelectronic device applications^19,20^. Besides improving the PCE value of the ST-OSC structure for practical applications, the average visible transmittance (AVT) is expected to be at least 25%^18,21,22^. Though the transparency of ST-OSC is improved in the optimization of the AVT values of the top contact material, its absorption may decrease below AM 1.5G. This occurs low photo-generated current density ($J_{{ph}}$) and a decrease in PCE values. With the increase of the thickness of active region, the $J_{{ph}}$ and PCE values of the ST-OSC have improved, while the optical characteristics and AVT values may seriously deteriorate. Therefore, there is a trade-off, which makes the optics inside the device more critical between photovoltaic performance and the visible transparency of the device. Appropriate selection of material and adjustion of its thickness are simultaneously optimized to achieve good conductivity and high AVT for the top electrode^7^.

For the purpose of semi-transparency in ST-OSC design, a thin metal material is sandwiched between two anti-reflection dielectrics as the top contact and a dielectric/metal/dielectric (DMD) structure is formed^23^. DMD designs offer high transparency and conductivity, low turbidity, excellent flexibility, easy fabrication and excellent compatibility with different substrates^24–36^. Therefore, it is clear that ST DMDs have a strong future, especially in ST optoelectronic devices^15,20,37–40^. An ST DMD electrode is designed to be able to fabricate ultra-thin metallic films and to improve the conductivity and flexibility of indium tin oxide (ITO). Recently, it has been used to replace ITO in SC applications^41^. DMD electrodes have taken a vital place in the design of OSC structures as ST. Even if the OSCs are grown over ITO, it is possible to make the opaque top contact transparent and the SC to become ST with the appropriate design of DMD structures^40^. It is noteworthy that DMD structures have simple fabrication processes and simple designs that do not require microscale or nanoscale shaping, such as photonic crystals^42,43^. Due to the inherently low reflectivity of the transparent electrodes, the photon absorption of the device must be carefully adjusted to allow sufficient light to pass through the device^18^.

In the present study, a MoO_3_/Ag/MoO_3_ transparent top contact in DMD structure was designed for opaque P3HT:PCBM-based OSCs in inverted structure architecture. We also studied the integration of DMD into OSC structure at optimal thickness values. In this context, we determined the optimal thickness values of both MoO_3_/Ag/MoO_3_ integrated into the FTO/ZnO/P3HT:PCBM/MoO_3_/Ag opaque-OSC structure and the current designed FTO/ZnO/P3HT:PCBM/MoO_3_/Ag/MoO_3_ ST-OSC structure based on the theoretical AVT and $J_{{ph}}$ values in the optical spectra calculated via transfer matrix method (TMM). We just focused on examination the CIE 1931 chromaticity coordinates of the structures depending on metal layer thickness. We optically characterized as well as the cell output parameters of ST-OSC designed and produced with optimal values were computed. Besides, a comparative evaluation of the calculated and experimantally obtained values was conducted.

## Results

The FTO/ZnO/P3HT:PCBM/MoO_3_/Ag opaque-OSC structure has Ag top electrode with a thickness of 100 nm. Ag has a very high reflectivity and low transmittance, especially in the VR. Thus, using Ag for top contact makes the OSC have opaque characteristics. We designed the ST-OSC by modifying the opaque Ag top contact of the structure using a MoO_3_/Ag/MoO_3_ DMD transparent top electrode with appropriate design parameters. In the DMD, the thickness of the inner dielectric MoO_3_ (*d*_*id*_), the thickness of the metal layer (*d*_*m*_) and the thickness of the outer dielectric MoO_3_ (*d*_*od*_) are parameters that considerably affect the optical characteristics of the OSC. Among these parameters, $d_{m}$ is a significant value defining the optical properties of the DMD and ST-OSC due to the optical characteristics of Ag. In addition, the inner MoO_3_ layer is directly integrated above the active layer P3HT:PCBM, so the layer also acts as the hole transport layer (HTL). The $d_{{id}}$ value can also influence output cell parameters, such as open-circuit voltage ($V_{{oc}}$), short-circuit current density ($J_{{sc}}$), maximum voltage ($V_{m}$), maximum current density ($J_{m}$), maximum power ($P_{m}$), PCE and fill factor (*FF*). Although the high thickness of the $d_{{id}}$ may adversely affect the electrical properties of the ST-OSC, it is known that it does not dramatically cause an impact on the optical properties, especially on AVT^44^. Since the photo-generated free carriers formed in the active area are swept to the contacts by the internal electric field, if the distance between the contacts and the active region is greater than the diffusion lengths, the free carriers may disappear by recombination. Therefore, the carriers cannot contribute to the current and cell output parameters may be adversely affected. Because the electrical contact is taken directly from the metal layer on which outer dielectric is located, the effects of $d_{m}$ and $d_{{od}}$ values on the ST-OSC output cell parameters are only possible by modifying the optical spectra of the structure.

For the reasons mentioned above, we first examined the effect of the $d_{m}$ value on optical properties in the design of DMD and ST-OSC. We made the design of DMDs integrated to the OSC by calculating optical spectra, such as transmittance and reflection spectra, and especially by evaluating the thickness parameters.

First, $d_{{id}}$ = 10 nm and $d_{{od}}$ = 30 nm have been set for the inner and outer MoO_3_ layers in TMM calculations, respectively. It is noticed that the $d_{{id}}$ value usually ranges between 1 and 10 nm^45–48^. For the $d_{{od}}$, we have chosen 30 nm because Chang et al. reported that the highest AVT value was observed for this value in MoO_3_/Ag/MoO_3_ structure^14^. Therefore, we took the chosen value of $d_{{od}}$ as a reference at the beginning of the calculations.

**Figure 1:**
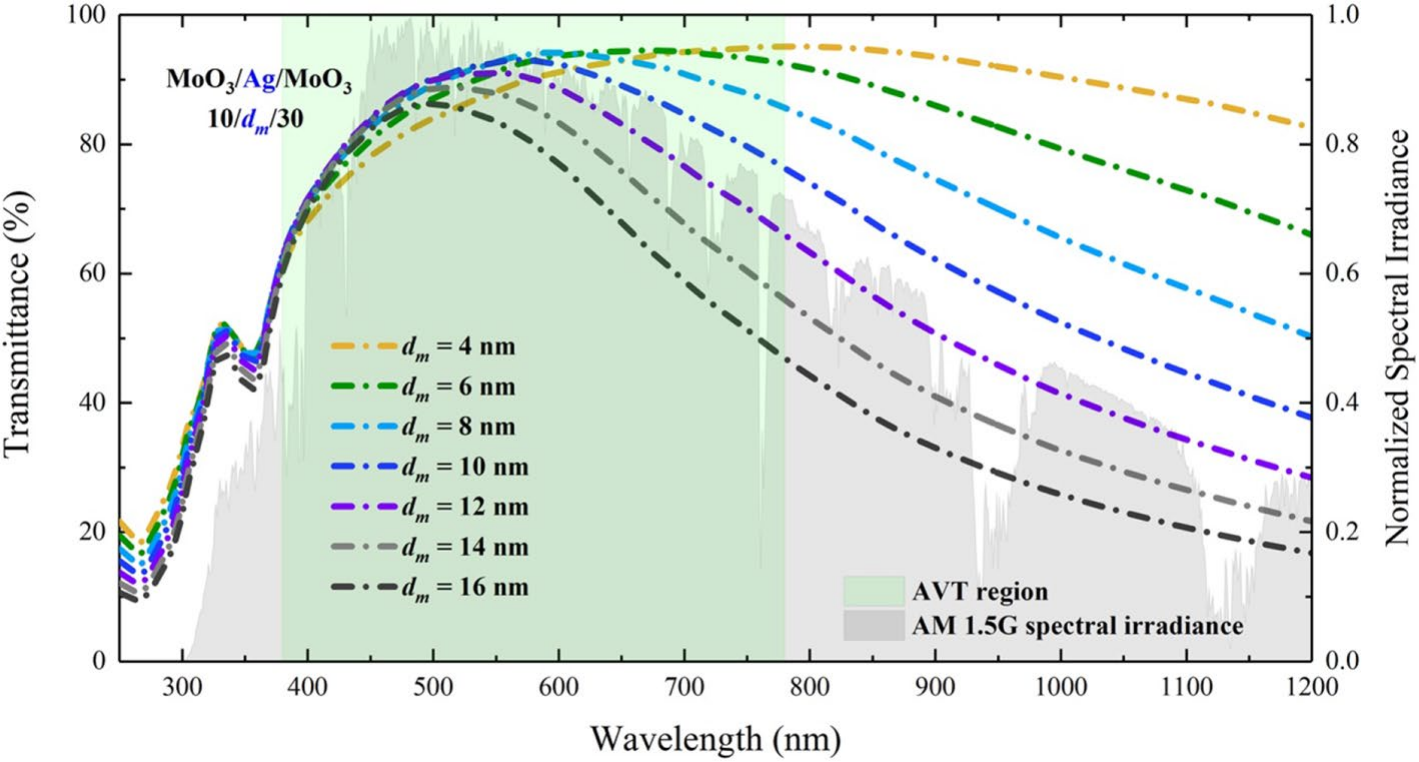
Calculated transmittance spectra of MoO_3_/Ag/MoO_3_ ($10\;{\text{nm}}/d_{m} /30~\;{\text{nm}}$) DMD transparent top contact. The green shaded area shows the region where AVT is calculated.

**Figure 2:**
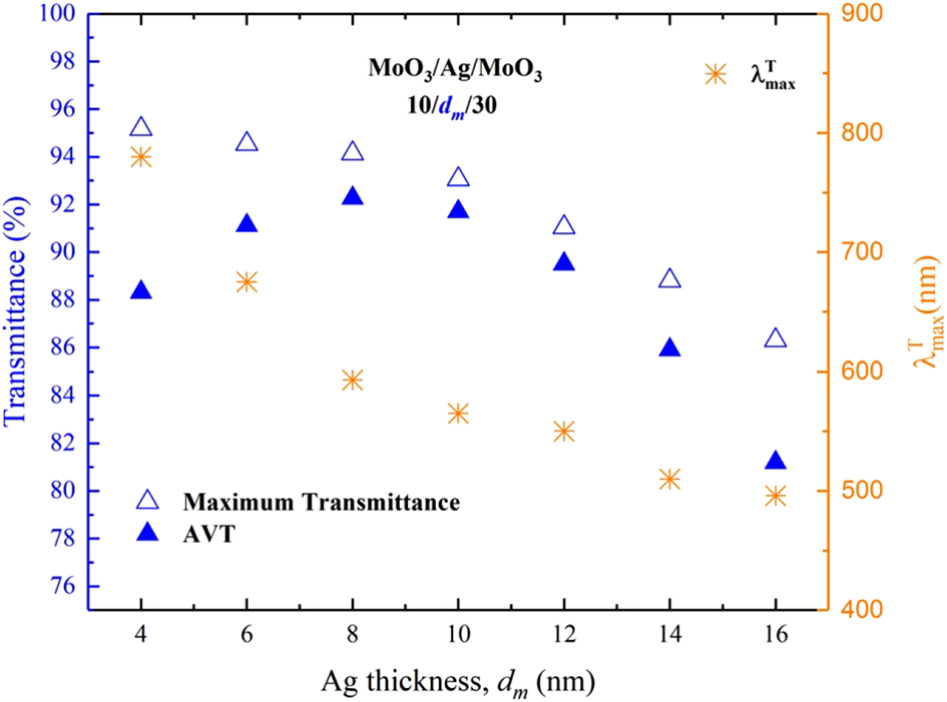
Changes of maximum transmittance, $\lambda _{{\max }}^{T}$ and AVT values of MoO_3_/Ag/MoO_3_ ($10\;{\text{nm}}/d_{m} /30\;{\text{nm}}$) DMD transparent top contact according to $d_{m}$.

The $d_{m}$ was changed between 4 and 16 nm with intervals of 2 nm, and we determined the effect of the Ag layer in DMD on the transmittance spectrum (Fig. 1). As seen in Fig. 1, for MoO_3_/Ag/MoO_3_ (10$/d_{m} /$30 nm), the transmittance is high for almost all investigated $d_{m}$ values, and we observed that the transmittance spectrum has the same trend with AM 1.5G spectral irradiance. This indicates that the DMD is a favourable transparent top electrode. Due to the high reflectivity of Ag at all wavelengths where AM 1.5G is responsible, increasing the $d_{m}$ value makes a decrease in the overall transmittance trend and especially in the maximum transmittance of the structure. When the DMD is integrated into ST-OSCs, the long wavelength of VR and the entire NIR will enhance the possibility of the re-absorption of unabsorbed electromagnetic waves as a result of the reflection of the electromagnetic waves to the active area. Thus, an improvement will occur in $J_{{ph}}$ and PCE values. Another remarkable result is that the wavelength value ($\lambda _{{\max }}^{T}$) at which the DMD transmittance spectrum has maximum transmittance decreased from 780 to 496 nm by varying $d_{m}$ from 4 to 16 nm (Fig. 2).

The transmittance characteristic of the ST-OSC with DMD is quite limited by the optical characteristics of the DMD. The AVT values of the ST-OSC will be related to the AVT values of the DMD. AVT values obtained from the calculated transmittance spectra of DMD are given in Fig. 2. For the thinnest Ag layer ($d_{m}$ = 4 nm), DMD has a maximum transmittance of 95.17%. This value is a result of the known reflectance and transmittance characteristics of Ag. However, there is an increment in AVT values from 88.32 to 92.26% with the change of $d_{m}$ from 4 to 8 nm. For $d_{m}$ > 8 nm, the AVT value tends to decrease to 81.19% (for $d_{m}$ = 16 nm). A maximum AVT value of 92.26% in DMD was found for $d_{m}$ = 8 nm. According to these results, Ag is an optimal structure to be used in ST-OSCs for DMD when $d_{m}$ is 8 nm.

**Figure 3:**
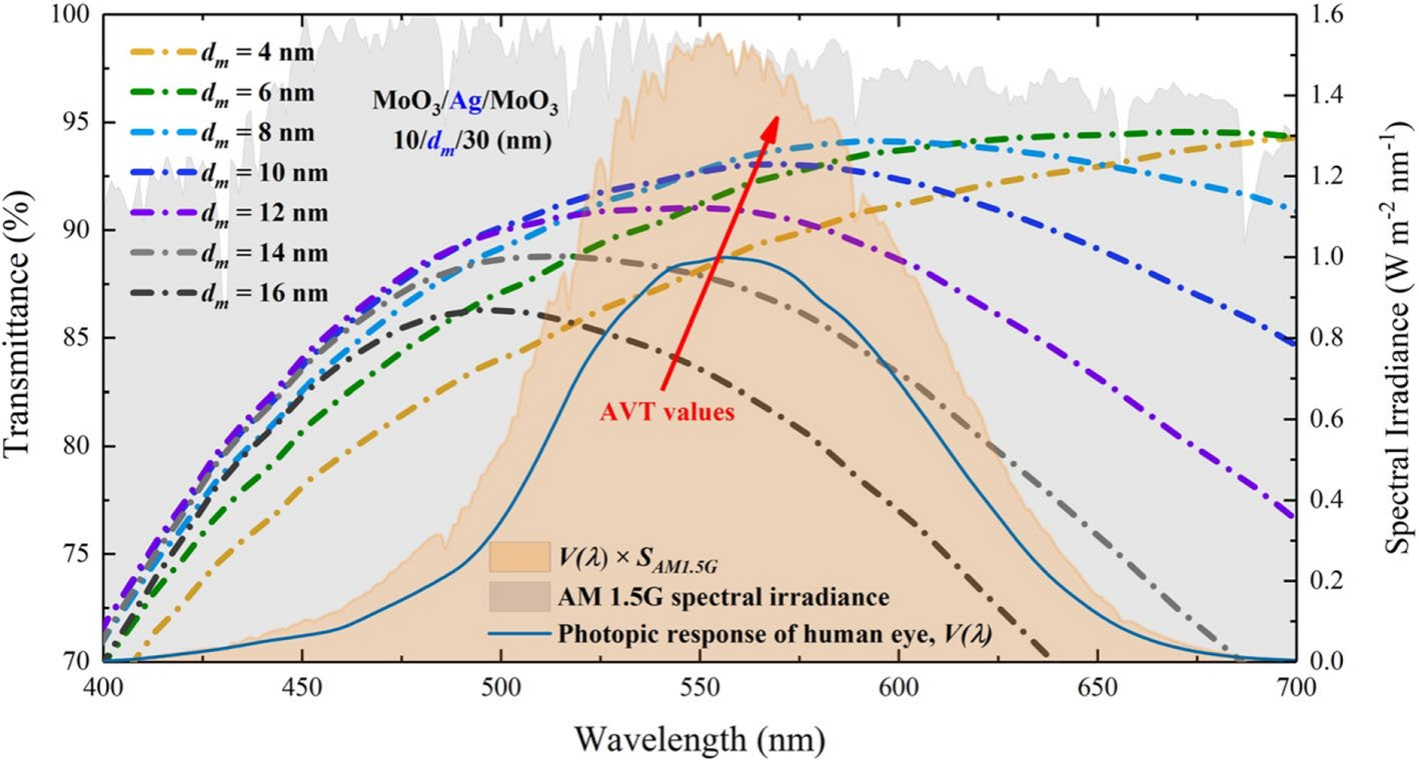
Analysis of the transmittance spectrum and AVT values of the MoO_3_/Ag/MoO_3_ ($10\;{\text{nm}}/d_{m} /30\;{\text{nm}}$) transparent top contact under $V(\lambda )$, AM 1.5G and $V(\lambda ) \times S_{{AM1.5G}} (\lambda )$ changes. The orange shaded area is related to the change of $V(\lambda ) \times S_{{AM1.5G}} (\lambda )$ and the area is the value of the integral in the denominator of Eq. (15).

$T_{{max}}$ value is not achieved for the structure that has the highest AVT. It is due to the fact that the AVT criterion is directly connected to $V(\lambda )$ and $S_{{AM1.5G}} (\lambda )$, as mentioned in Eq. (16). It should not always be expected that the AVT value of an optimal structure with high optical transmittance is high. It should be taken into consideration that the selectivity and response of the human eye for different wavelengths are also important in perception for window applications. As seen in Fig. 3, the mismatch between transmittance and AVT relationship in DMD can be noticed.

The wavelength range of the human eye is sensitive in the range of 400–700 nm, and Eq. (16) includes $V(\lambda ) \times S_{{AM1.5G}} (\lambda )$ multiply both the numerator and denominator. Therefore, even though the integral boundaries are over VR of the electromagnetic spectrum, integrants have values in the 400–700 nm range (Fig. 3). As seen in Fig. 3, it belongs to the structure of $d_{m}$ = 8 nm with the highest transmittance value within the orange hatched area representing the $V(\lambda ) \times S_{{AM1.5G}} (\lambda )$ expression, and the AVT of this structure is also the highest. The maximum transmittance is observed for $d_{m}$ = 4 nm in the DMD, whereas the highest AVT value is obtained for $d_{m}$ = 8 nm. The red arrow orientation in Fig. 3 indicates the variation of AVT. In the light of all these explanations, the transmittance characteristic of a structure does not directly give information about its AVT; an analysis as in Fig. 3 provides a more accurate AVT assessment.

The DMD produced with $d_{m}$ = 8 nm, which belongs to the highest AVT, is very suitable for ST optoelectronic devices. However, the optical characteristics of the optoelectronic device to be produced with this DMD cannot be designed based only on the detailed examination for MoO_3_/Ag/MoO_3_. This is because in the TMM calculations made for MoO_3_/Ag/MoO_3,_ the modelling is made with electromagnetic waves coming perpendicularly to the surface. However, the presence of the DMD in ST optoelectronic devices should be re-evaluated because the electromagnetic wave can follow different paths as a result of reflection, refraction or absorption in different regions until it reaches the DMD by passing through the OSC. The angle and condition in which the components of the electromagnetic wave reach the inner dielectric surface should also be taken into account. Thus, TMM calculations should be made for the entire structure of the optoelectronic device, and the optimal structure parameters should be determined.

**Figure 4:**
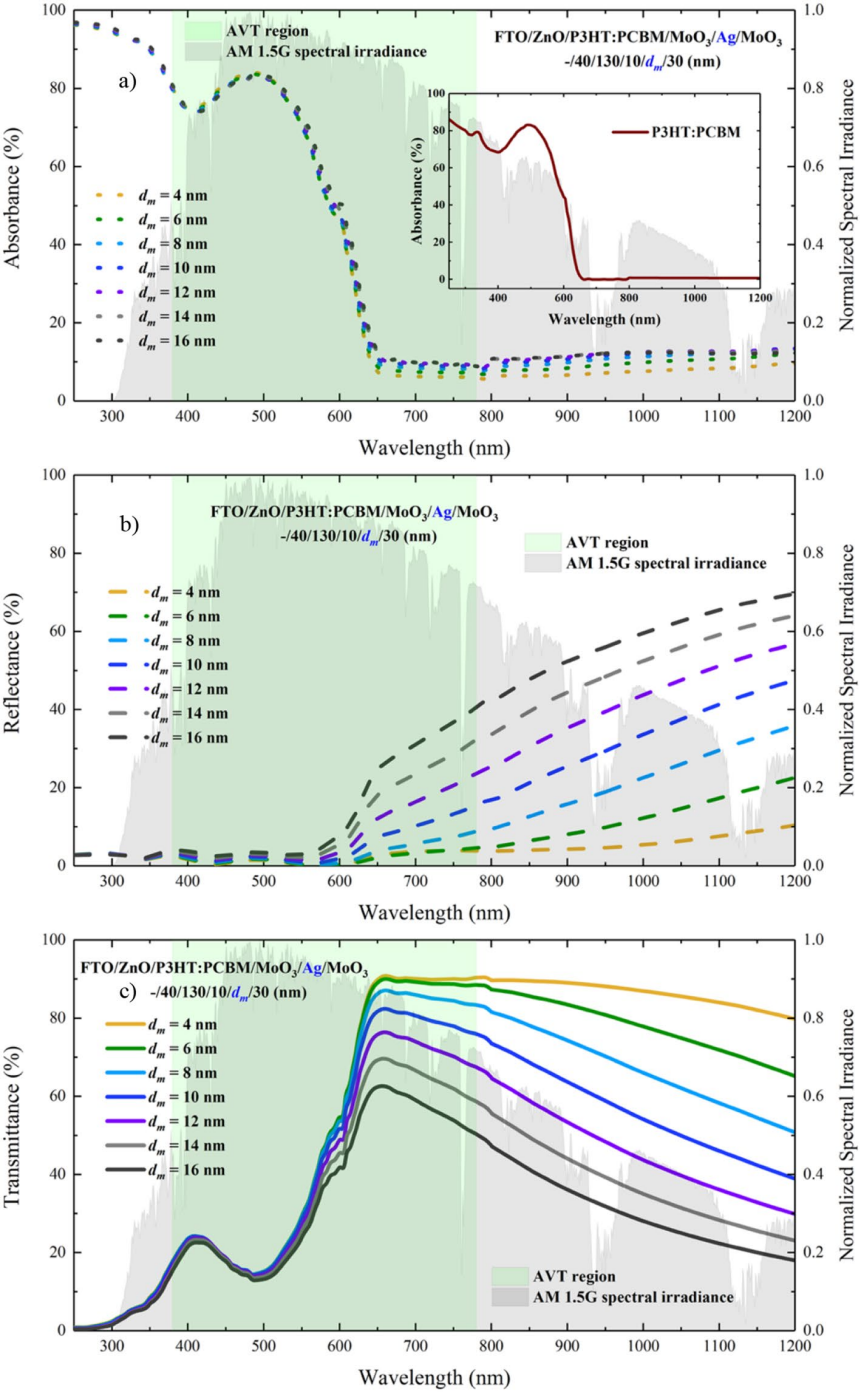
For FTO/ZnO/P3HT:PCBM/MoO_3_/Ag/MoO_3_ (–/40/130/10/*d*_*m*_/30 nm) ST-OSC; (**a**) absorbance, (**b**) reflectance and (**c**) transmittance spectra. (**a**) Shows the absorbance spectrum of the P3HT:PCBM polymer blend that forms the active area of the inset ST-OSC.

We performed the optical characterization of the ST-OSC structure formed by the integration of the MoO_3_/Ag/MoO_3_ (10/$d_{m}$/$d_{{od}}$ nm) instead of the Ag. We calculated the optical characteristics of FTO/ZnO/P3HT:PCBM/MoO_3_/Ag/MoO_3_ (–/40/130/10/$d_{m}$/30 nm) ST-OSC according to the $d_{m}$ change by setting $d_{{id}}$ = 10 nm and $d_{{od}}$ = 30 nm. The calculated absorbance, reflectance and transmittance spectrum are shown in Fig. 4a–c, respectively. The characteristic of the absorbance spectrum of the ST-OSC structure is the same of which the P3HT:PCBM for all wavelengths (in Fig. 4a as an inset).

**Figure 5:**
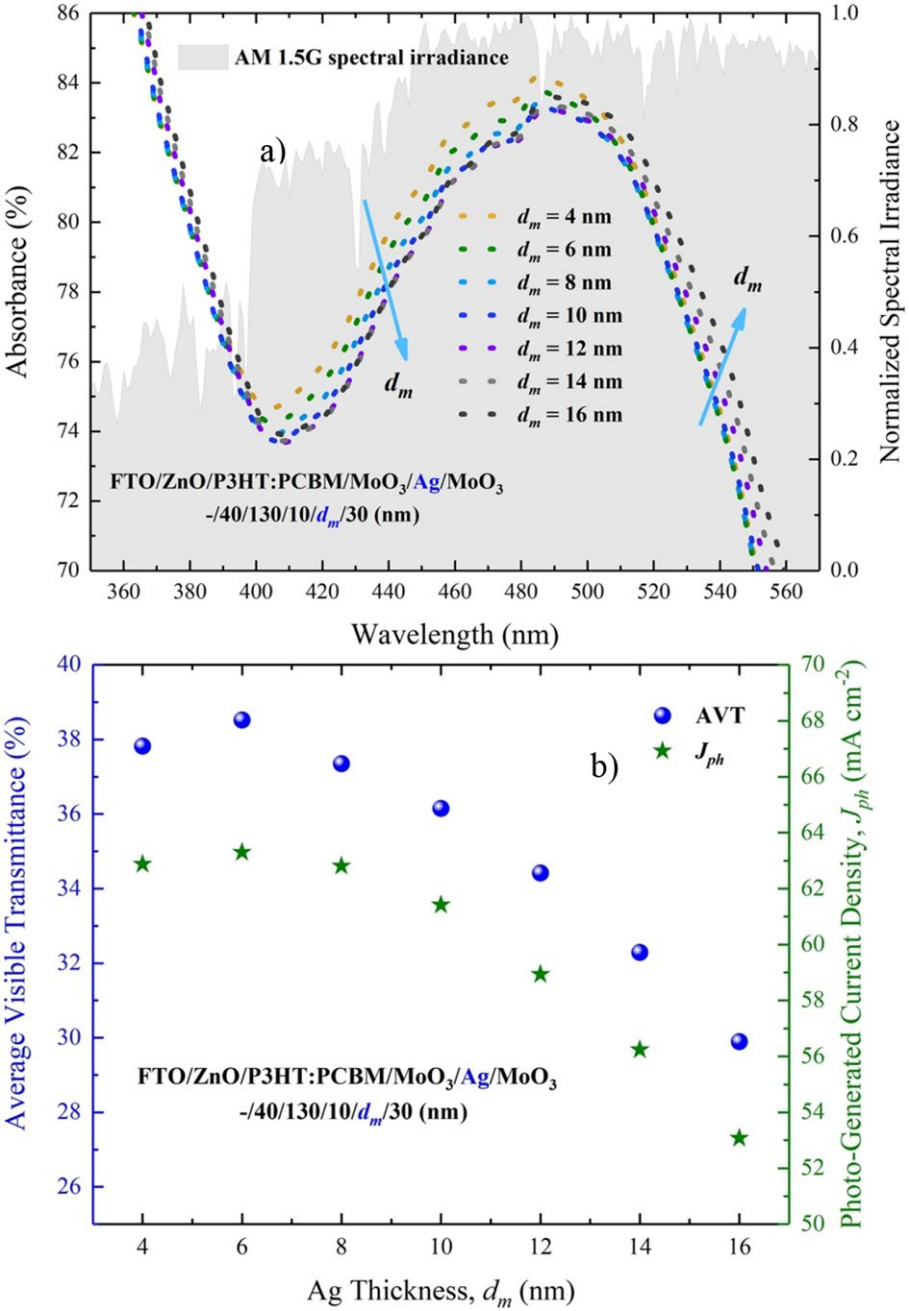
For FTO/ZnO/P3HT:PCBM/MoO_3_/Ag/MoO_3_ (–/40/130/10/*d*_*m*_/30 nm) ST-OSC; (**a**) absorbance spectrum and, (**b**) changes of AVT and $J_{{ph}}$ according to $d_{m}$.

With the change of the $d_{m}$ value, an enhancement occurs in the absorbance and reflectance of the ST-OSC in VR (longer than 560 nm) and NIR. While this rate of change is especially effective in the 4–10 nm range of $d_{m}$ for absorption, it loses its effectiveness after $d_{m}$ = 10 nm (Fig. 4b). Increasing the $d_{m}$ allows non–absorbed photons to be sent back into the structure in the active region and can advance the $J_{{ph}}$ value. However, to make a more effective interpretation for $J_{{ph}}$, the AM 1.5G spectral irradiation should also be considered. An improvement in absorption occurs with the increase of $d_{m}$ in the ST–OSC (Fig. 5a). Therefore, to better examine the effect of $d_{m}$ on $J_{{ph}}$, a calculation should be made in the entire wavelength range of the AM 1.5G spectral irradiation for $J_{{ph}}$. The $J_{{ph}}$ is calculated as follows:

1 $$J_{{ph}} = \mathop \smallint \limits_{{\lambda _{{low}} }}^{{\lambda _{{up}} }} AM1.5G(\lambda )~T(\lambda )~\left( {1 - e^{{ - \alpha (\lambda )d_{{al}} }} } \right)~d\lambda$$

where $\alpha (\lambda )$ and $d_{{al}} ~$ are the absorption coefficient and thickness of P3HT:PCBM, respectively. The $J_{{ph}}$ change calculated based on $d_{m}$ is given in Fig. 5b.

We calculated the highest $J_{{ph}}$ value for ST-OSC as 63.30 mA/cm^2^ for $d_{m}$ = 6 nm. The $d_{m}$ adversely effects to $J_{{ph}}$ in accordance decreasing absorption in the region where AM 1.5G spectral irradiance is high (Fig. 5a). We calculated AVT values, which are very important for ST-OSC, by using Eq. (16) (Fig. 5b). The highest AVT value of 38.52% was found for $d_{m}$ = 6 nm. By reaching $d_{m}$ = 16 nm, the AVT value decreasing 29.89% is even higher than 25%, which is the lower limit accepted for window applications. This result pointed out that the DMD designed at optimal values for P3HT:PCBM-based ST-OSCs is an appropriate contact.

**Figure 6:**
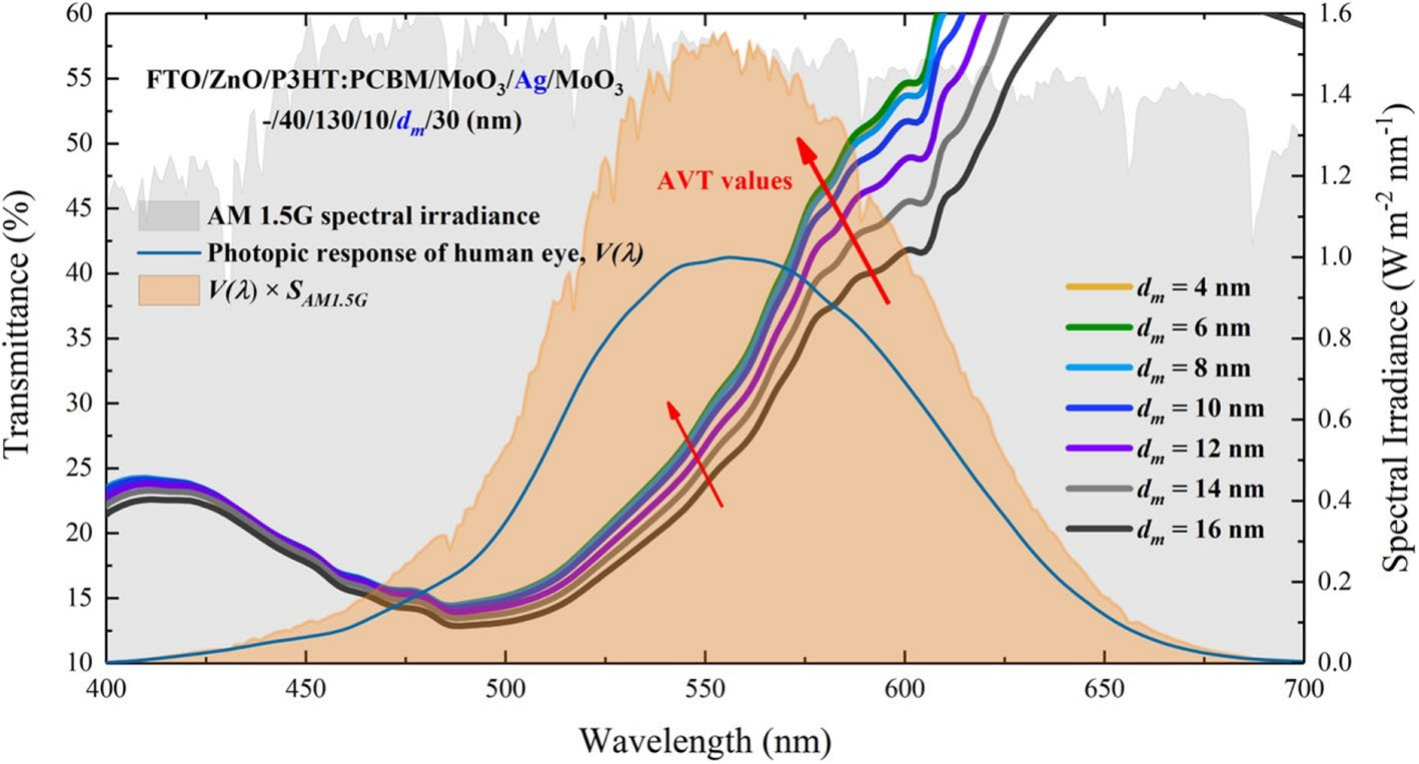
Transmittance spectra of FTO/ZnO/P3HT:PCBM/MoO_3_/Ag/MoO_3_. (–/40/130/10/$d_{m}$/30 nm) ST-OSC according to $d_{m}$ and analysis of AVT values under $V(\lambda )$, AM 1.5G and $V(\lambda ) \times S_{{AM1.5G}} (\lambda )$ changes. The orange shaded area is related to the change of $V(\lambda ) \times S_{{AM1.5G}} (\lambda )$ and the area is the value of the integral in the denominator of Eq. (15).

**Figure 7:**
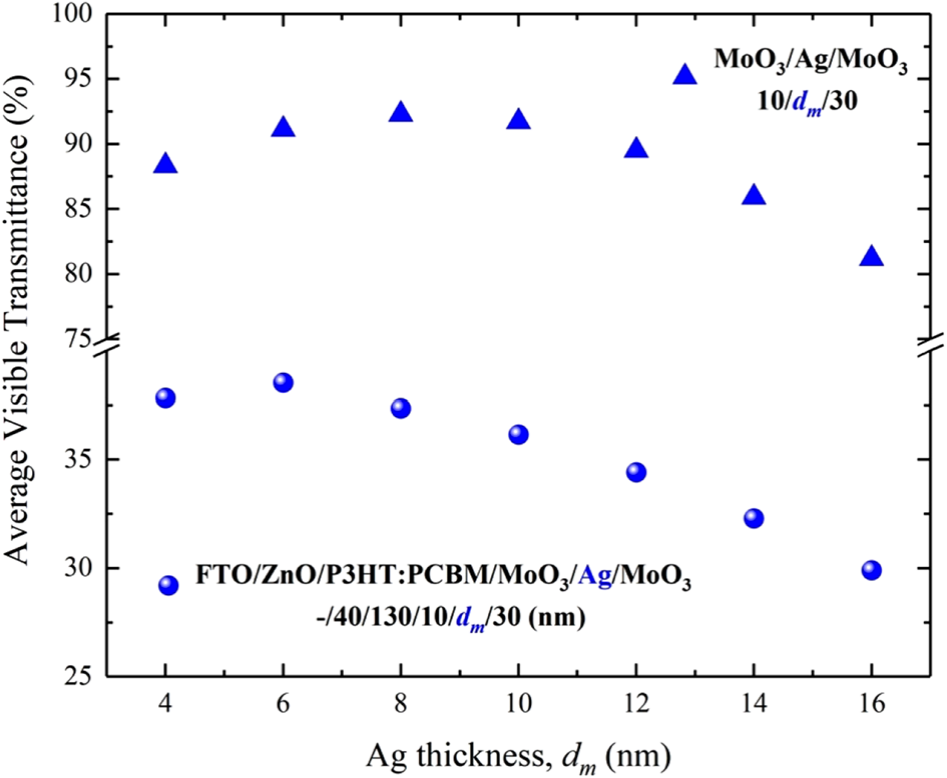
Comparative representation of the FTO/ZnO/P3HT:PCBM/MoO_3_/Ag/MoO_3_ (–/40/130/10/$d_{m}$/30 nm) ST-OSC and AVT values according to $d_{m}$ of MoO_3_/Ag/MoO_3_ (10/$d_{m}$/30 nm).

The $d_{m}$ behavior did not cause a serious change in the transmittance spectra for the wavelength region less than 500 nm. However, as seen in Fig. 4c, the increase in $d_{m}$ caused a serious decrease in VR (longer than 500 nm) and NIR due to the reflective characteristics of Ag in the transmittance spectrum. AVT values are not directly influenced by these variations. The AVT value obtained for $d_{m}$ = 4 nm is 37.82%, whereas the AVT value for $d_{m}$ = 6 nm is 38.52% (Figs. 6 and 7). These values can be realized when the integral arguments in Fig. 6 and Eq. (16) are examined. No matter how much the transmittance varies during AM 1.5G, the values of the structures in the regions where the $V(\lambda ) \times S_{{AM1.5G}} (\lambda )$ product intersects determine the changes in AVTs (Fig. 6). Within the orange hatched area representing the expression of $V(\lambda ) \times S_{{AM1.5G}} (\lambda )$, the highest transmittance value belongs to the structure with $d_{m}$ = 6 nm, and the AVT of this structure is at the highest value. The red arrow orientation in Fig. 6 indicates the variation of AVT.

When the ST-OSC is designed for $d_{m}$ = 6 nm, it is noticeable as the most suitable structure in terms of both $J_{{ph}}$ and AVT. However, the highest AVT value of the DMD was obtained for $d_{m}$ = 8 nm. The AVT values obtained for both ST-OSC and DMD are comparatively given in Fig. 7. This inconsistency in the AVT, which occurs by simply investigating the DMD and integrating the DMD into the OSC, can be explained by the fact that the electromagnetic wave, which reaches the DMD structure, follows different paths due to reflection, refraction or absorption in different regions. In this case, it is an effective way to consider reaching the surface in which the angle and condition of the components of the electromagnetic wave that reflected, absorbed and eventually reached the DMD in the OSC.

Even with $d_{m}$ reaching up 10 nm, there is a significant decrease of 22.3% in the AVT values. This result indicated that Ag has a remarkable effect on the optical characteristics of the ST-OSC.

**Figure 8:**
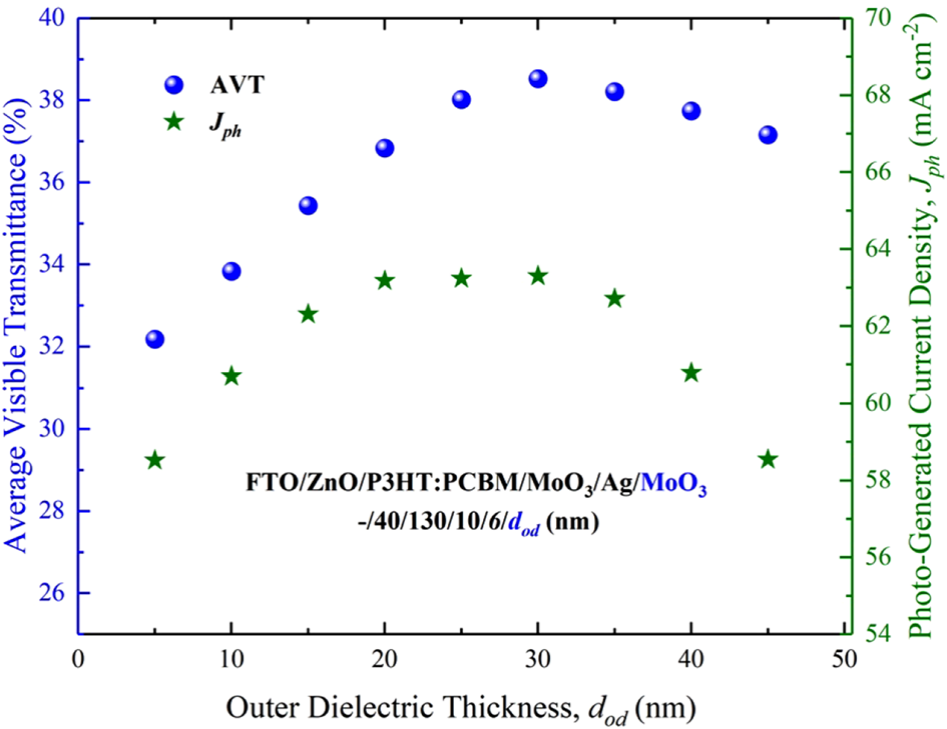
AVT and $J_{{ph}}$ changes of FTO/ZnO/P3HT:PCBM/MoO_3_/Ag/MoO_3_. (–/40/130/10/6/$d_{{od}}$ nm) ST-OSC according to $d_{{od}}$.

We determined that the optimal value for Ag was $d_{m}$ = 6 nm. Thus, to obtain a more detailed evaluation, we analyzed the effect of the $d_{{od}}$, on the AVT and $J_{{ph}}$ values of the ST-OSC. The changes of the FTO/ZnO/P3HT:PCBM/MoO_3_/Ag/MoO_3_ (–/40/130/10/6/$d_{{od}}$ nm) ST-OSC in $d_{{od}}$, AVT and $J_{{ph}}$ are given in Fig. 8.

As seen in Fig. 8, for the ST-OSC structure, although the change of $d_{{od}}$ from 5 to 30 nm seems to cause a saturation in the AVT values, there is a slight decrease in the AVT values from 30 to 45 nm. The highest AVT value was obtained for $d_{{od}}$ = 30 nm, and the value is 38.52%. Depending on $d_{{od}}$, this characteristic change in the AVT indicated that a high AVT value could be achieved for the ST-OSC structure designed for $d_{{od}}$ ≥ 30 nm. Although the $J_{{ph}}$ value showed an improvement in $d_{{od}}$ up to 20 nm, it was saturated after this value. We obtained the maximum $J_{{ph}}$ of 63.30 mA/cm^2^ for $d_{{od}} ~$ = 30 nm. Even if AVT was still high in ST-OSCs designed for $d_{{od}} \ge ~$ 30 nm, there was a serious decrease in $J_{{ph}}$.

Considering the highest AVT and $J_{{ph}}$ values for the ST-OSC, we obtained the structure with optimal values for $d_{m}$ = 6 nm and $~d_{{od}}$ = 30 nm. Therefore, within the scope of the study, the investigated ST-OSC was produced at these values.

In addition, the $d_{{od}} ~$ parameter does not affect the AVT of the ST-OSC as much as the Ag layer. Therefore, we examined the effect of Ag, which is the dominant layer in optical characteristics, on CIE 1931 chromaticity coordinates in ST-OSC.

When examining the colour coordinates, AM 1.5G’s coordinates are $x$ = 0.32020 and $y$ = 0.33240, and D65’s colour coordinates are $x$ = 0.3128 and $y$ = 0.3290. In the design of ST-OSCs with neutral colours for window applications, it is generally expected in order to get colour coordinates at the achromatic point ($x$ = 0.3333 and $y$ = 0.3333) or equal to the coordinates of AM 1.5G or D65^7^. In addition, the colour coordinates can be controlled with transmittance spectra by the modification of the structure design. Thus, window applications with different colours can be obtained as a result of producing ST-OSCs in the desired colour.

**Figure 9:**
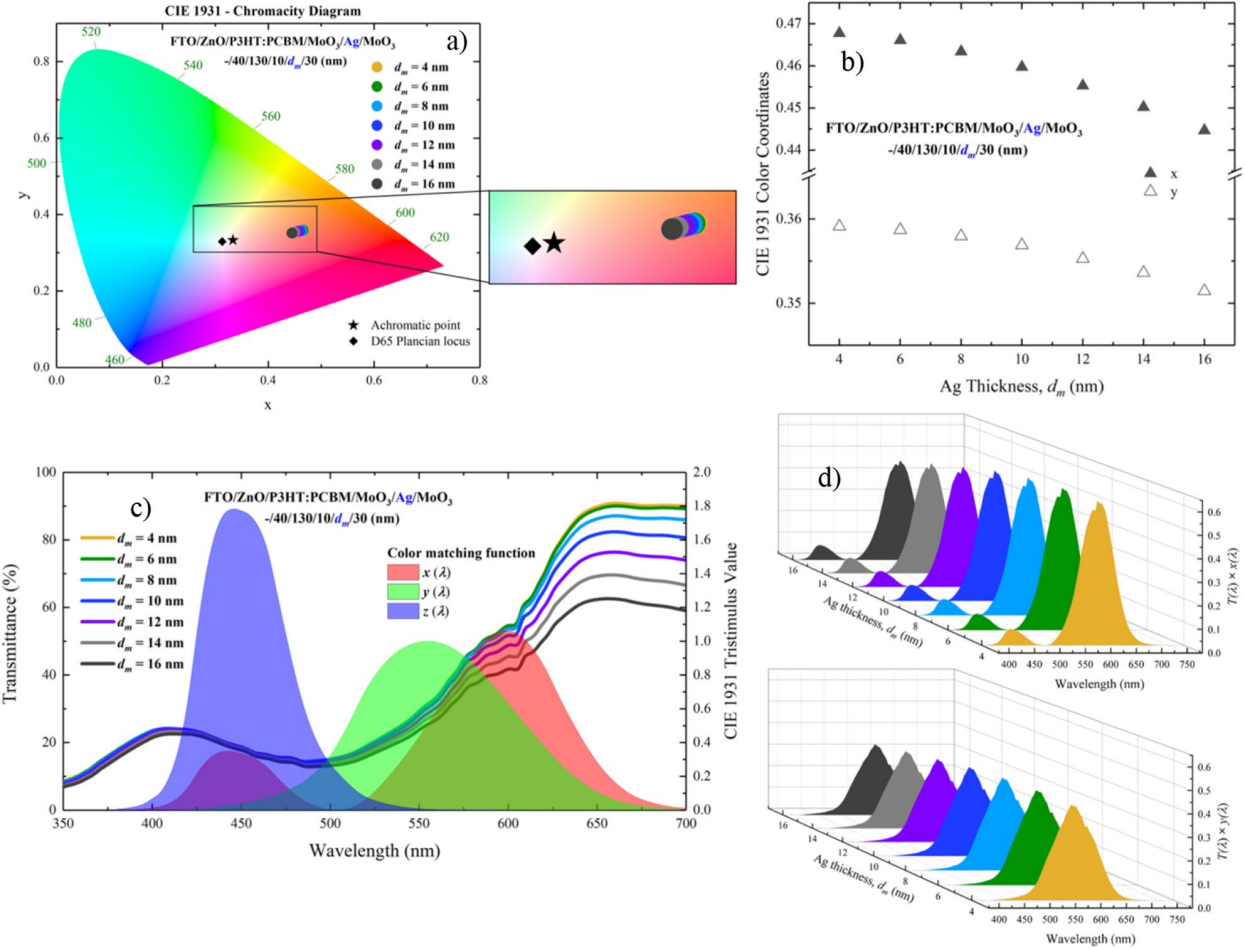
Calculations for FTO/ZnO/P3HT:PCBM/MoO_3_/Ag/MoO_3_ (–/40/130/10/$d_{m}$/10 nm) ST-OSC with different $d_{m}$; (**a**) the location of the color coordinates in the CIE 1931 chromaticity diagram, (**b**) the change of color coordinates, (**c**) the intersection of the transmittance characteristic of the ST-OSC with the $\bar{x}(\lambda )$, $\bar{y}(\lambda )$, $\bar{z}(\lambda )$ color matching functions, and (**d**) the change of $T(\lambda )~\bar{x}(\lambda )$ ve $T(\lambda )~\bar{y}(\lambda )$ functions.

According to the $d_{m}$ changes of the ST-OSC, the locations and changes of the colour coordinates of the ST-OSC in the CIE 1931 chromaticity diagram under AM 1.5G illumination are given in Fig. 9a,b, respectively. It was clearly seen from the CIE chromaticity diagram that the change of $d_{m} ~$ from 4 to 16 nm shifted the colour coordinates of the ST-OSC towards the D65 Planckian locus. The colour coordinates shifted from 0.468 to 0.445 for $x$ and from 0.359 to 0.351 for $y$. We observed this change, which Ag makes in the colour coordinates of the ST-OSC, especially in *x* in Fig. 9c,d. As given in Fig. 9c, by increasing $d_{m}$, the transmittance of the ST-OSC in the region where it intersects with $\bar{x}(\lambda )$ decreased more than in the region where it intersects with $\bar{y}(\lambda )$. Therefore, the change in *x* would be greater. The effect of this change in transmittance was directly modified. Thus, the $T(\lambda )~\bar{x}(\lambda )$ and $T(\lambda )~\bar{y}(\lambda )$ factors appear in the integrants in Eqs. (17) and (18). The variation of the areas under the $T(\lambda )~\bar{x}(\lambda )$ and $T(\lambda )~\bar{y}(\lambda )$ factors with $d_{m}$ directly presented the changes of *x* and *y* colour coordinates, respectively (Fig. 9d).

**Figure 10:**
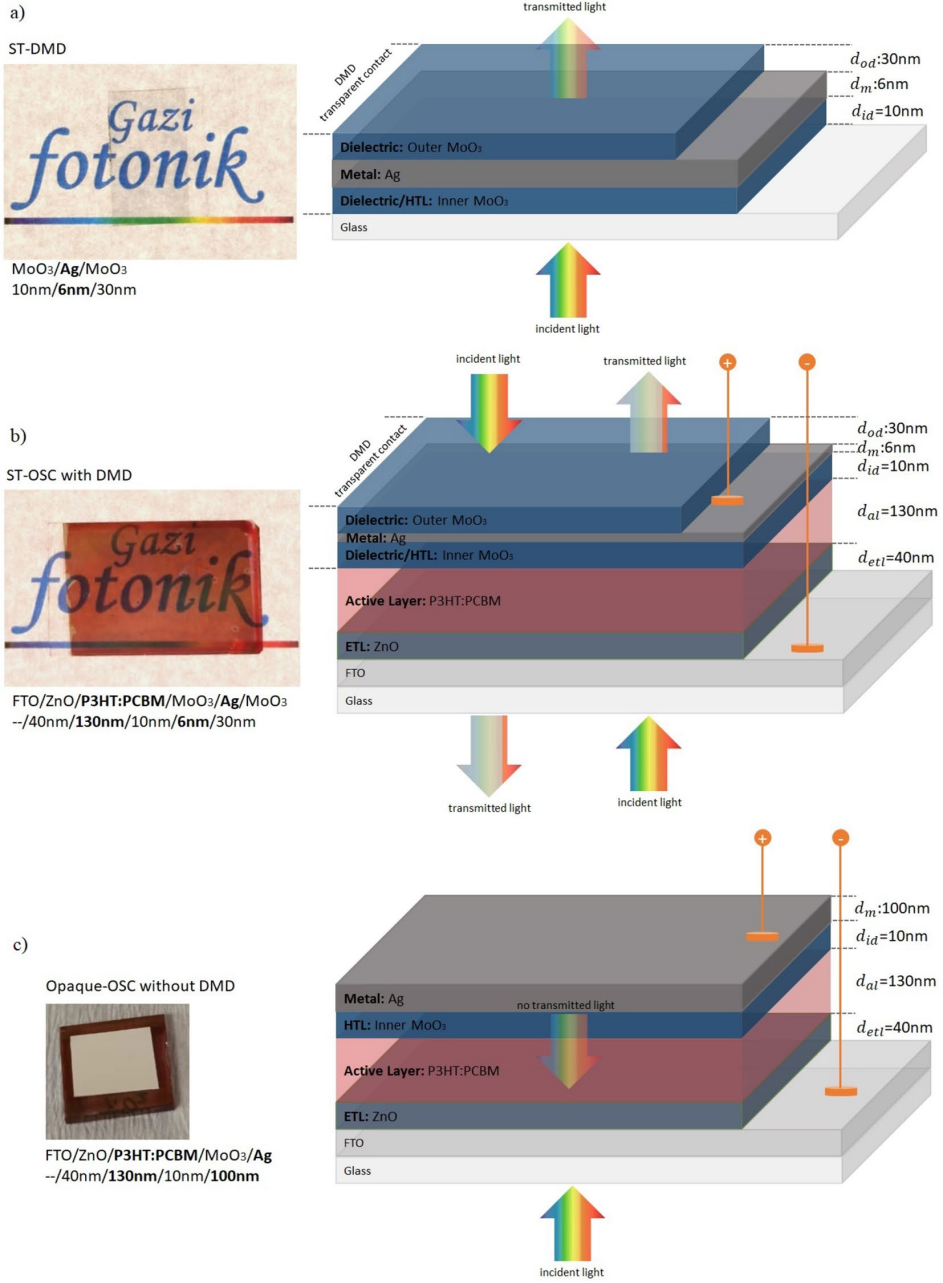
The photo and structure of; (**a**) the MoO_3_/Ag/MoO_3_ (10/6/30 nm) transparent top contact, (**b**) FTO/ZnO/P3HT:PCBM/MoO_3_/Ag/MoO_3_ (–/40/130/10/6/30 nm) ST-OSC, (**c**) FTO/ZnO/P3HT:PCBM/MoO_3_/Ag (–/40/130/10/100 nm) opaque-OSC.

The CIE 1931 colour coordinates of ST-OSC for $d_{m}$ = 6 nm and $d_{{od}}$ = 30 nm with the highest AVT and $J_{{ph}}$ were 0.4661 and 0.3587 for the *x* and *y* values, respectively. The schematic representation and photographs of the DMD, ST-OSC and opaque-OSC are given in Fig. 10a–c, respectively.

**Figure 11:**
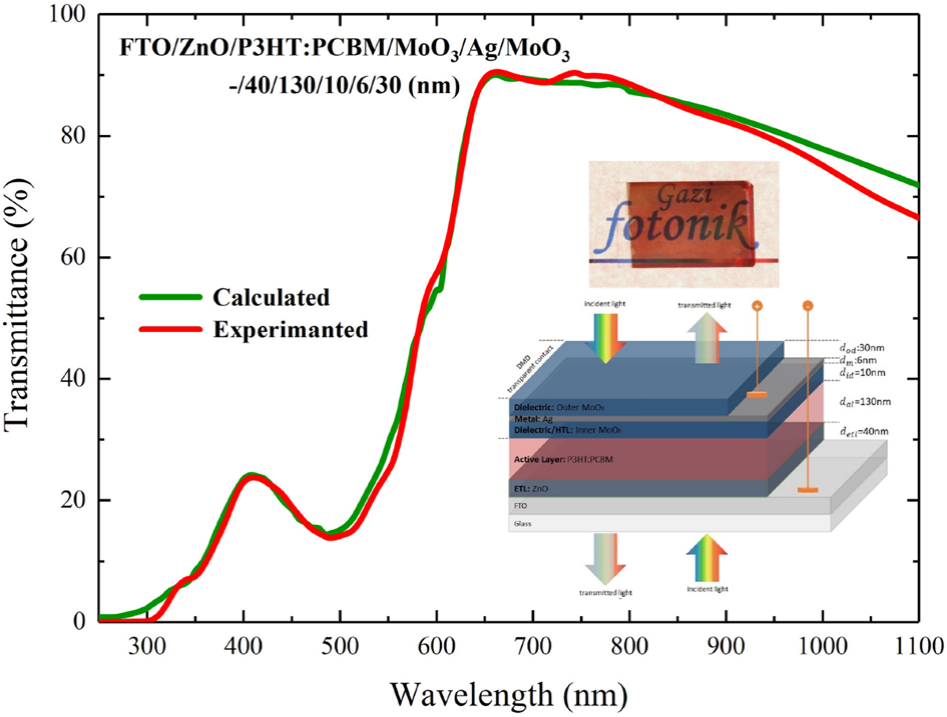
Calculated and experimental transmittance spectra of FTO/ZnO/P3HT:PCBM/MoO_3_/Ag/MoO_3_ (–/40/130/10/6/30 nm) ST-OSC.

As seen in Fig. 10a, the top contact is quite transparent, and its colour selectivity is high. The photograph of the ST-OSC given in Fig. 10b indicates that the transmittance was high for all VR, and the colour selectivity was low in the red region of VR. This selectivity is not surprising according to the transmittance calculations made with TMM for this structure. The high transmittance in the calculated and experimental transmittance spectrum of the ST-OSC given in Fig. 11, especially for wavelengths higher than 550 nm, led us to determine the colour of the ST-OSC as red. On the other hand, it was seen from the photograph of the opaque-OSC produced with $d_{m}$ = 100 nm in Fig. 10c that the structure was completely reflective but not transmittant. Especially for VR, electromagnetic waves entering by the glass are reflected back into the structure by the Ag layer, and the structure has opaque characteristics.

The calculated and experimental values of AVT obtained from the transmittance spectrum for the ST-OSC (Fig. 11). In addition, the *x* and *y* values obtained from the calculated transmittance spectrum and experimental transmittance spectrum for the CIE 1931 colour coordinates are given in Table 1.

**Table 1:** AVT values and CIE 1931 color coordinates of FTO/ZnO/P3HT:PCBM/MoO_3_/Ag/MoO_3_ (–/40/130/10/6/30 nm) ST-OSC.

	AVT (%)	CIE 1931 color coordinates	
		*x*	*y*
Calculated	38.52	0.4661	0.3587
Experimental	37.42	0.4613	0.3500

**Figure 12:**
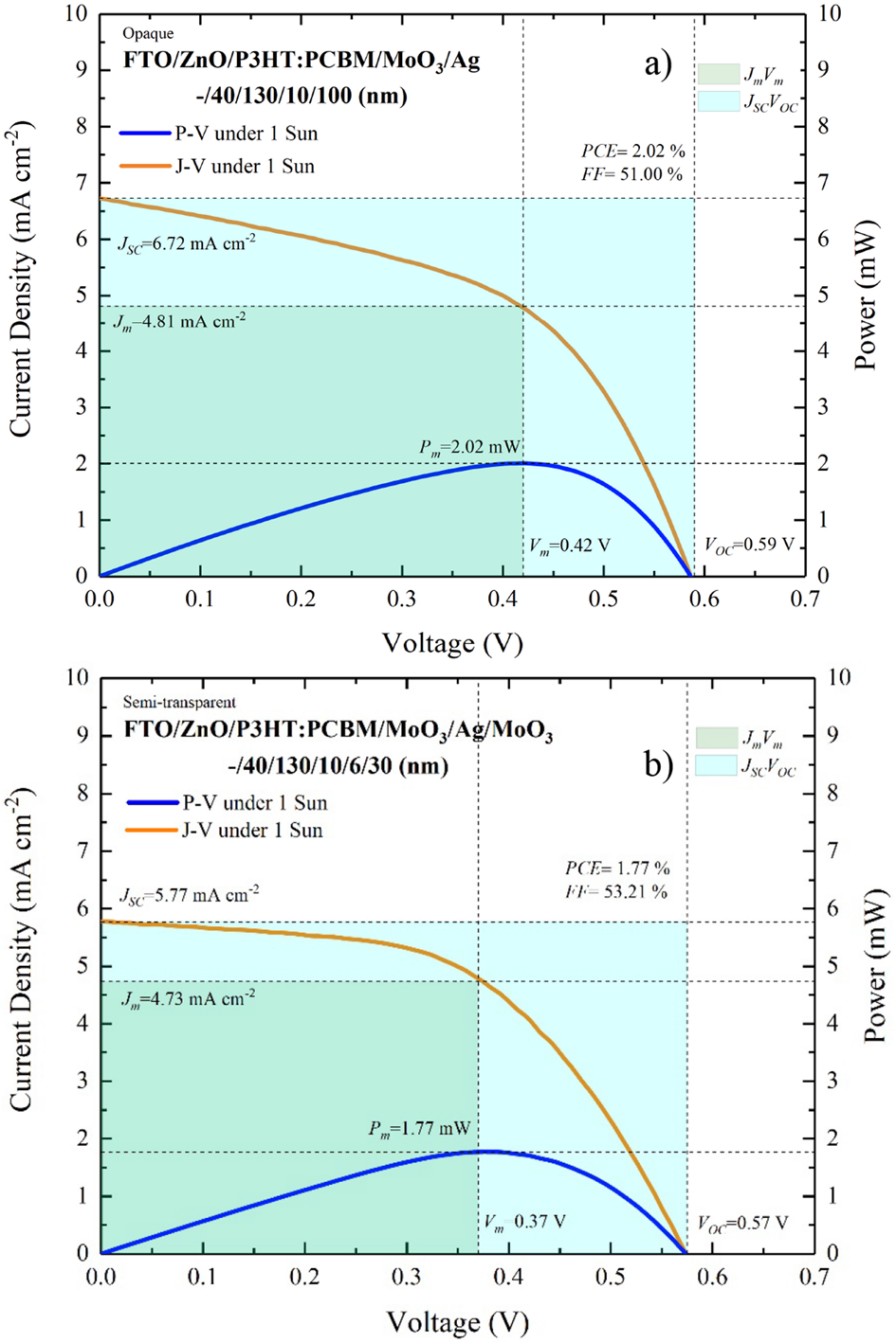
$~J - V$ characteristics of (**a**) FTO/ZnO/P3HT:PCBM/MoO_3_/Ag (–/40/130/10/100 nm) opaque-OSC and (**b**) FTO/ZnO/P3HT:PCBM/MoO_3_/Ag/MoO_3_ (–/40/130/10/6/30 nm) ST-OSC.

*J*–*V* measurements of the opaque-OSC produced with $d_{m}$ = 100 nm and of the ST-OSC produced for $d_{m}$ = 6 nm and $d_{{od}}$ = 30 nm are given in Fig. 12a,b, respectively. The cell output parameters of both structures are given in Table 2. *FF* values for both structures are above 50%. The thickness of the MoO_3_ ($d_{{id}}$ = 10 nm) layer serving as HTL in opaque and ST structures was the same, and there was no significant change in the $V_{{oc}}$, $J_{m}$ and $V_{m}$ values. However, the $J_{{sc}}$ value was higher in the opaque structure compared to the ST structure. This can be explained by the re-absorption as a result of the reflection of photons, which entered the glass in the opaque-OSC and were not absorbed in the active layer, back into the structure from the opaque Ag. Thanks to the DMD in the ST-OSC, low reflectivity and high transmittance do not support the re-absorption process and a decrease in $J_{{sc}}$ occurred.

**Table 2:** The cell output parameters of (a) FTO/ZnO/P3HT:PCBM/MoO_3_/Ag (–/40/130/10/100 nm) opaque-OSC and, (b) FTO/ZnO/P3HT:PCBM/MoO_3_/Ag/MoO_3_ (–/40/130/10/6/30 nm) ST-OSC.

	$J_{{sc}}$ (mA cm^−2^)	$J_{m}$ (mA cm^−2^)	$V_{{oc}}$ (V)	$V_{m}$ (V)	$P_{m}$ (mW)	*FF* (%)	PCE (%)
ST-OSC	5.77	4.73	0.57	0.37	1.77	53.21	1.77
Opaque-OSC	6.72	4.81	0.59	0.42	2.02	51.00	2.02

Although a slightly lower efficiency of ST-OSCs compared to opaque-OSCs is a disadvantage for a device that converts electromagnetic energy into electrical energy, reduction in the efficiency is typically negligible for window applications with ST structure or when it is considered that OSC is obtained in different colours. Determining the optimal values for ST-OSC that works with the principle of photon absorption and allows the propagation of photons in the region that it absorbs is very important for electronic and optoelectronic applications.

## Discussion

The main motivation of the study is to design and to produce a ST-OSC device. In designation process,we have presented that the MoO_3_/Ag/MoO_3_ DMD structure is a suitable top transparent contact for P3HT:PCBM-based OSCs by determining the optimal values of $d_{m}$ and $d_{{od}}$. The fact that the transmittance values obtained for $d_{m}$ in the range of 4–16 nm are quite high supports that the transmittance spectrum of DMD has the same trend as AM 1.5G spectral irradiance. Increasing the $d_{m}$ value is enhanced the absorption and reflection of the ST-OSC in VR (wavelength above 560 nm) and NIR, indicating a trend of decrease in the transmittance spectrum. Furthermore, it can be concluded that the maximum transmittance of the ST-OSC structure is also reduced, since a significant decrease in the $\lambda _{{\max }}^{T}$ value of the DMD transmittance spectrum from 780 to 496 nm is observed.

The structure with $d_{m}$ = 6 nm (with highest value of $J_{{ph}}$) and $d_{{od}}$ = 30 nm is an optimal structure to be used in ST-OSCs by considering the AVT value is taken as a reference for window applications. We reported that the increment of $d_{m}$ shifted the colour coordinates of the ST-OSC towards the D65 Planckian locus.

We have observed that no considerable change in the cell output parameters such as $V_{{oc}}$, $J_{{sc}}$ as well as *FF* for both the opaque and ST-OSC structures produced. It is possible that the computed results of the parameters can said to be in good agreement with the results in the literature containing OSCs with an active layer of P3HT:PCBM^20,45^.

Considering that the ST structure has a valuably utilization in window applications or OSCs obtained in different colours, the modification of opaque-OSC as the ST structure obviously make the decrease in PCE value negligible. Moreover, it is emphasized that we have obtained an effectively working ST-OSC in optoelectronic device applications.

## Material and methods

### Calculation of optical characteristics

We performed the calculations by using the TMM, which is a highly effective method used in the simulations of optoelectronic devices. TMM is one of the important methods that analyze how the electromagnetic wave propagates within the structure and theoretically determine the optical characteristics of the structure, especially the DMD^49–51^. The position of the electric and magnetic field components within the DMD or OSC can be determined by a transfer matrix and propagation matrix^49^. While the electric and magnetic field components of the electromagnetic wave are connected to each other with a transfer matrix at each interface of the DMD layers, the spreading field components in the DMD are connected to each other by the propagation matrix.

**Figure 13:**
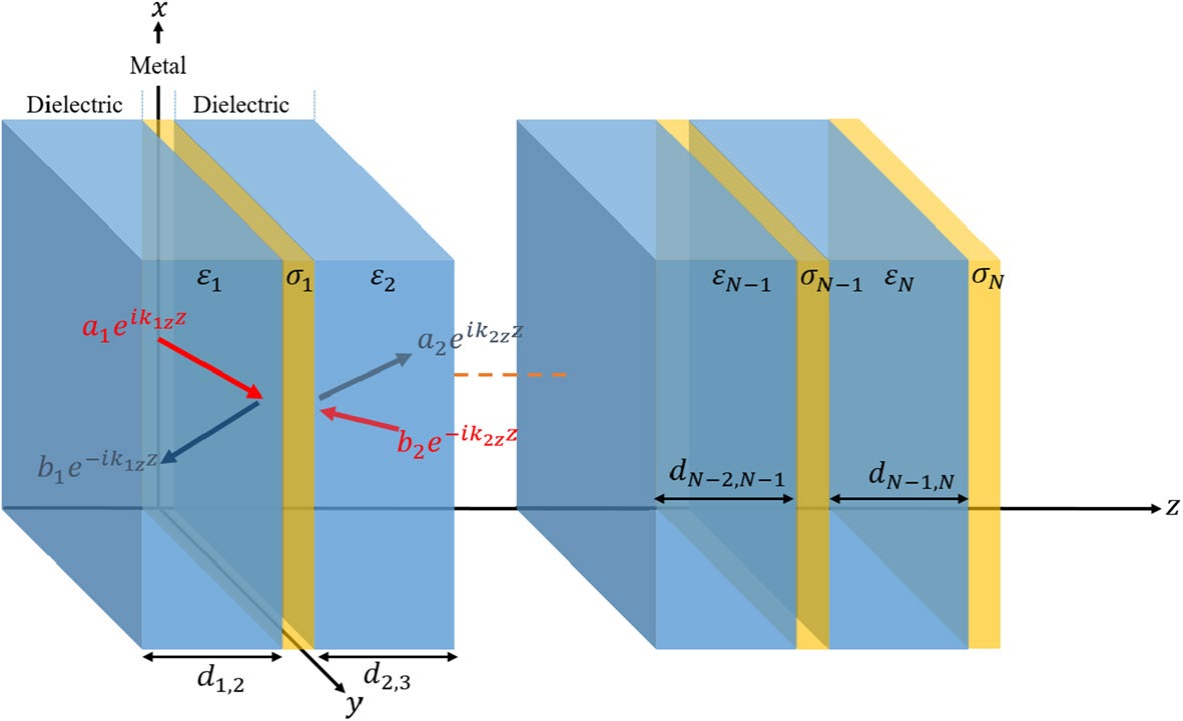
A thin metal layer with σ conductivity surrounded by two dielectric layers with values $\varepsilon _{1}$ and $\varepsilon _{2}$. The structure is self-repeatable and can be reduced to a DMD structure for a single metal layer. Red and blue arrows represent the electromagnetic wave at the interface and at the reflected, respectively.

As seen in Fig. 13, the metal layer surrounded by two dielectrics for the DMD structure has a conductivity ($\sigma$) distributed parallel to the $z = 0~$ planes. It is necessary to examine the propagation of the electromagnetic wave on the interfaces of the conductor with the dielectrics. Assuming that the electromagnetic wave is polarized in the $y$ direction and propagates in the $z$ direction, the $s$ and $p$ polarizations can be examined. The magnetic field for polarization *p* can be written in the following form:

2 $$H_{{1y}} = \alpha _{1} e^{{i\vec{k}_{1} .\vec{r}}} + \beta _{1} e^{{i\vec{k}_{1} .\vec{r}}} = \left( {\alpha _{1} e^{{ik_{{1z}} z}} + \beta _{1} e^{{ - ik_{{1z}} z}} } \right)e^{{ - ik_{{1x}} x}} ,~~\quad z < 0$$

3 $$H_{{2y}} = \alpha _{2} e^{{i\vec{k}_{2} .\vec{r}}} + \beta _{2} e^{{i\vec{k}_{2} .\vec{r}}} = \left( {\alpha _{2} e^{{ik_{{2z}} z}} + \beta _{2} e^{{ - ik_{{2z}} z}} } \right)e^{{ - ik_{{2x}} x}} ,~~\quad z > 0$$

where $\vec{k}_{i} = \sqrt {\varepsilon _{i} } \omega /c\;~(i = 1,~\;2)$ is the wave vector of the electromagnetic wave, $\varepsilon _{i} \;~(i = 1,~\;2)$ is the dielectric constant of the medium, $\omega$ is the angular frequency, $c$ is the propagation velocity of the electromagnetic wave in space and $\alpha _{i}$ and $\beta _{i} ~(i = 1,\;2)$ are the coefficients. According to Snell’s law, the $x$ components of the wave vector in both media at the interface will be equal to each other: $k_{{1x}} = k_{{2x}}$. If we also apply the boundary conditions of the electric field and the magnetic field for the interface^52^, we obtain the equations found below:

4 $$\left. {\hat{n}_{s} \times (\vec{E}_{2} - \vec{E}_{1} )} \right|_{{z = 0}} = 0$$

5 $$\left. {\hat{n}_{s} \times (\vec{H}_{2} - \vec{H}_{1} )} \right|_{{z = 0}} = \vec{J}$$

Here, $\hat{n}_{s}$ is the normal unit vector of the surface, and $\vec{J}$ is the surface current density of the metallic layer. In addition, by obtaining $\vec{J}$ by Ohm’s law and applying the $z = 0$ condition, the following equations are obtained:

6 $$\frac{{k_{{1z}} }}{{\varepsilon _{1} }}(a_{1} - b_{1} ) - \frac{{k_{{2z}} }}{{\varepsilon _{2} }}(a_{2} - b_{2} ) = 0$$

7 $$(a_{1} + b_{1} ) - (a_{2} + b_{2} ) = J_{x}$$

8 $$J_{x} = \left. {\sigma E_{x} } \right|_{{z = 0}} = \frac{{\sigma k_{{2z}} }}{{\varepsilon _{0} \varepsilon _{2} \omega }}(a_{2} - b_{2} )$$

where $\varepsilon _{0}$ is the permittivity of the space. By combination of Eqs. (6), (7) and (8), $a_{i}$ and $b_{i}$ ($i = 1$) can be associated with $a_{{i + 1}}$, $b_{{i + 1}}$ and the transition matrix $M_{{i \to i + 1}}$.

9 $$\left( {\begin{array}{*{20}c} {a_{i} } \\ {b_{i} } \\ \end{array} } \right) = M_{{i \to i + 1}} \left( {\begin{array}{*{20}c} {a_{{i + 1}} } \\ {b_{{i + 1}} } \\ \end{array} } \right)$$

The $M_{{i \to i + 1}}$ transition matrix is $M_{{1 \to 2}}$ as follows when $~i = 1$ is selected for DMD design with a single metallic layer:

10 $$M_{{1 \to 2}} = \frac{1}{2}\left( {\begin{array}{*{20}c} {1 + n_{p} + \xi _{p} } & {\quad 1 - n_{p} - \xi _{p} } \\ {1 - n_{p} + \xi _{p} } & {\quad 1 + n_{p} - \xi _{p} } \\ \end{array} } \right)$$

where $n_{p} = \frac{{\varepsilon _{1} k_{{2z}} }}{{\varepsilon _{2} k_{{1z}} }}$, and $\xi _{p} = \frac{{\sigma k_{{2z}} }}{{\varepsilon _{0} \varepsilon _{2} \omega }}$. The above magnetic field and related boundary conditions for polarization $p$ and all processes performed by the application of Ohm’s law are also applicable to the electric field component for *s* polarization. In this case, again by using Ohm’s law and boundary conditions, the transition matrix of *s* polarization is obtained as follows:

11 $$M_{{1 \to 2}} = \frac{1}{2}\left( {\begin{array}{*{20}c} {1 + n_{s} + \xi _{s} } & {1 - n_{s} + \xi _{s} } \\ {1 - n_{s} - \xi _{s} } & {1 + n_{s} - \xi _{s} } \\ \end{array} } \right)$$

where $\mu _{0}$ is the transmittance of the space, the parameters $n_{s}$ and $\xi _{s}$ are equal to the terms $\frac{{k_{{2z}} }}{{k_{{1z}} }}$ and $\frac{{\sigma \mu _{0} \omega }}{{k_{{1z}} }}$, respectively. The $n_{p}$ and $n_{s}$ terms used in the derivation of the equations are directly related to the refractive index of the layers and include the absorption coefficient within the complex term. These values depend on the wavelength and the angle of incidence of the electromagnetic wave.

Considering the equations, the transition matrices obtained for both $s$ and $p$ polarizations are the same, except for the sign difference in non-diagonal components. By making the necessary arrangements for $j = (s,\;p)$ and $\eta _{p} = 1$, $~\eta _{s} = - 1$, a common transition matrix can be parameterized as follows:

12 $$M_{{1 \to 2}} = \frac{1}{2}\left( {\begin{array}{*{20}c} {1 + n_{j} + \xi _{j} } & {\quad 1 - n_{j} - \eta _{j} \xi _{j} } \\ {1 - n_{j} + \eta _{j} \xi _{j} } & {\quad 1 + n_{j} - \xi _{j} } \\ \end{array} } \right)$$

The relationship between the transition ($t$) and reflection ($r$) coefficients of the electromagnetic wave at the interfaces with the transition matrix as well as the transmittance ($T$) and reflection ($R$) spectrum of the DMD and ST-OSC structures can be calculated as follows:

13 $$R = ~\left| r \right|^{2} = \left| {\frac{{M_{{2 \to 1}} }}{{M_{{1 \to 1}} }}} \right|^{2}$$

14 $$T = ~\left| t \right|^{2} = \left| {\frac{1}{{M_{{1 \to 1}} }}} \right|^{2}$$

In addition, obtaining the $T$ and $R$ spectrum of the structures enables the absorption ($A$) spectrum to be obtained by the following:

15 $$A = 1 - (T + R)$$

### Determination of average visible transmittance

The transparency properties of ST-OSCs are determined by both AVT values and by transmittance characteristics in the visible light wavelength range (370–740 nm), taking into account the photonic response of the human eye ($V~(\lambda )$). The AVT value is calculated by the following formula^7,53^:

16 $$AVT = \frac{{\mathop \int \nolimits_{{370~\;{\text{nm}}}}^{{780~\;{\text{nm}}}} T(\lambda )~V(\lambda )~S_{{AM1.5G}} (\lambda )~d\lambda }}{{\mathop \int \nolimits_{{370~\;{\text{nm}}}}^{{780~\;{\text{nm}}}} V(\lambda )~S_{{AM1.5G}} (\lambda )~d\lambda }}$$

where $S_{{AM1.5G}} (\lambda )$ is the photon flux under AM 1.5G illumination (Fig. 3). AVT values depend on the working environment of ST-OSCs, and an AVT value of 25% is an acceptable criterion for window applications^7,21^. In addition, if an experimental result is to be obtained in AVT calculation, the T (λ) value to be used should be experimental, and the beam spot must be within the effective area during the measurement. If the spot area is larger than the effective area, some of the incident light may directly reach the detector and experimentally create an error in the transmittance measurement^7,54^.

### Evaluation of color coordinates of ST-OSC

Another characteristic of ST-OSCs as important as the AVT is the colour coordinates ($x$*,* $y$) in the CIE 1931 chromaticity diagram. The diagram is designed based on the response of the human eye and is often used to determine the colour characteristics of illuminators^7^. For ST-OSCs, the colour coordinates of the OSC were determined using the CIE 1931 chromaticity diagram under AM 1.5G illumination^55^. In the tristimulus system, $X$, $Y$ and $Z$ values can be calculated under VR with the following equations:

17 $$X = \mathop \int \limits_{{370\;{\text{nm}}}}^{{780\;{\text{nm}}}} S_{{AM1.5G}}^{{D65}} (\lambda )~T(\lambda )~\bar{x}(\lambda )\;~~d\lambda$$

18 $$Y = \mathop \int \limits_{{370~\;{\text{nm}}}}^{{780~\;{\text{nm}}}} S_{{AM1.5G}}^{{D65}} (\lambda )~T(\lambda )~\bar{y}(\lambda )\;d\lambda$$

19 $$Z = \mathop \int \limits_{{370~\;{\text{nm}}}}^{{780\;{\text{nm}}}} S_{{AM1.5G}}^{{D65}} (\lambda )~T(\lambda )~\bar{z}(\lambda )~\;d\lambda$$

In all these equations, $S_{{AM1.5G}}^{{D65}}$ is the CIE standard D65 illuminant spectrum, and the terms $\bar{x}(\lambda ),\;~~\bar{y}(\lambda )$ and $\bar{z}(\lambda )$ are colour-matching functions defined by the CIE protocol $(X + Y + Z) = 1$ and the colour coordinates can be simplified to two-dimensional coordinates^56^:

20 $$x = \frac{X}{{(X + Y + Z)}}$$

21 $$y = \frac{Y}{{(X + Y + Z)}}$$

### Experimental details

**Figure 14:**
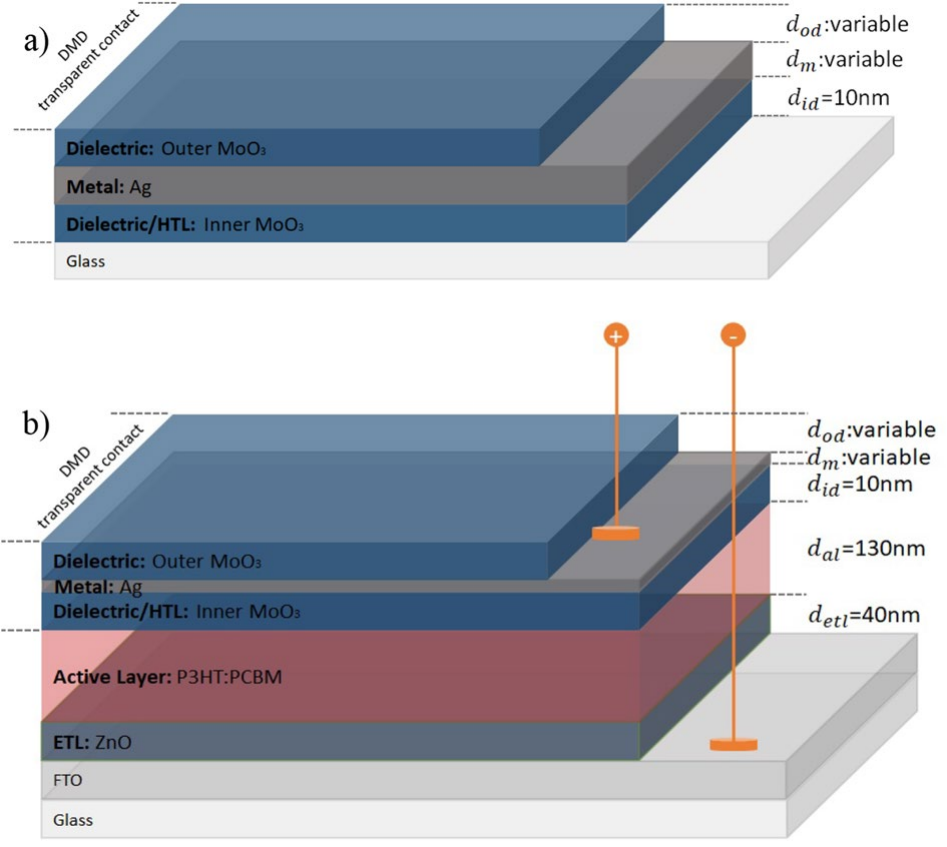
Schematic representation of (**a**) MoO_3_/Ag/MoO_3_ DMD transparent top contact and, (**b**) FTO/ZnO/P3HT:PCBM/MoO_3_/Ag/MoO_3_ ST-OSC.

In the scope of study, ST-OSCs and opaque-OSCs have the active region, which is the blend of poly 3-hexylthiophene-2, 5-diyl (P3HT) and poly 6, 6-phenyl C61-butyric acid methyl ester (PCBM). Except for the structure and design of upper electrodes, all structure parameters are the same. The structure of OSC, active region and top transparent contacts are formed as inverted device, bulk-heterojunction and DMD, respectively (Fig. 14a,b). All layers in the OSC structures, except the active region, were coated using the Nanovak NVTS500 Sputtering system.

When used as the bottom electrode, fluorine tin oxide (FTO) coated on glass has a high optical transmittance (about 90%) and low work function (~ 4.4 eV) as well as being electrically conductive^57^. Before SC production, we cleaned glass substrates coated with FTO of organic and inorganic contaminants. In the cleaning process, we ultrasonically immersed the substrates in detergent and deionized water for 5 min, and then rinsed with distilled water. We then cleaned the samples with pure acetone and isopropyl alcohol for 10 min in different containers in an ultrasonic bath and again rinsed them with deionized water. In the last process, we dried FTO substrates with high-purity nitrogen (N_2_) gas and made a metal oxide coating.

We used a ZnO layer as ETL due to its high optical transmittance in the VR and NIR regions and high carrier mobility (0.066 cm^2^ V^−1^ s^−1^)^58–60^. We made the deposition of ZnO with the sputtering technique using a ZnO target with a purity of 99.999%. To get high optical transmittance, the thickness $(d_{{etl}} )~$ of ETL was set to 40 nm. We coated the active region of SCs with the spin coating technique using P3HT:PCBM. We prepared the solution from the ready-made P3HT and PCBM polymers by blending them evenly by means of mass and adding dichlorobenzene solvent at a concentration of 20 mg/mL. We allowed this to mix homogeneously with a magnetic stirrer for 14 h at 80 °C. For all OSCs, we set P3HT:PCBM active layer thickness ($d_{{al}}$) as 130 nm. We used MoO_3_ as HTL. For the produced opaque-OSC, the MoO_3_ layer acts exactly as an HTL. However, this situation differs for ST-OSC in that MoO_3_ forms both the HTL and the inner part in the DMD. The HTL layer prevents a possible penetration of the metal of the DMD for ST-OSC or the metal for opaque-OSC towards the active layer. It also functions as an electron-blocking layer in its structures. We determined the thickness of the HTL layer ($d_{{htl}} = d_{{id}}$) in all structures as 10 nm. We created the Ag for opaque-OSC using the 100 nm thick by sputtering technique. We produced MoO_3_/Ag/MoO_3_ for ST-OSC using the sputtering technique with $d_{{id}}$ = 10 nm and optimal $d_{m}$ and $d_{{od}}$ thicknesses as determined by our calculations. The high purity (99.999%) of MoO_3_ and Ag targets were used.

We determined the optical characteristics of the produced structures with a Perkin Elmer Lambda 2S UV–Vis–NIR spectrometer in the 300–1100 nm wavelength range. In addition, we used the Keithley 4200 source meter for current density–voltage (*J*–*V*) measurements and the Newport Oriel-Sol1A solar simulator for AM 1.5G illumination.

## Data Availability

The datasets generated during and/or analysed during the current study are available from the corresponding author (Ç.Ç.) on reasonable request.
